# From structural code to immunomodulation: the structure-activity relationship, molecular mechanisms, and translational medicine challenges of yam (*Dioscorea opposita* Thunb) polysaccharides

**DOI:** 10.3389/fimmu.2026.1821913

**Published:** 2026-05-14

**Authors:** Xiaoling Ding, Bo Wang, Xin Ma, Shan He, Noundara, Xuebing Zhou

**Affiliations:** People’s Hospital of Ningxia Hui Autonomous Region, Ningxia Medical University, Yinchuan, China

**Keywords:** clinical translation, immune regulation, structure activity relationship, TLR4 signaling pathway, yam polysaccharides

## Abstract

Chinese yam (*Dioscorea opposita* Thunb), a traditional medicinal and edible plant, has attracted significant attention for its bioactive components. Chinese yam polysaccharides (CYPs) are regarded as the principal bioactive fraction responsible for its pleiotropic effects, notably immunomodulation. This review systematically summarizes recent advances in understanding the chemical structure, immunomodulatory mechanisms, and structure-activity relationships of CYPs. A core emphasis is placed on the intimate correlation between immunologic activity and specific structural determinants, including molecular weight, monosaccharide profile, glycosidic linkages, and higher-order conformation. Mechanistic studies demonstrate that CYPs are recognized by pattern recognition receptors, primarily TLR4, on the surface of immune cells such as macrophages. This recognition activates pivotal downstream signaling pathways, including NF-κB and MAPK. The subsequent orchestration of cytokine networks enables a homeostatic regulation of both innate and adaptive immunity, characterized by context-dependent immunostimulation and anti-inflammatory potential. Chemical modifications like sulfation and selenylation, alongside bioprocessing strategies such as physical, enzymatic, and fermentation treatments, significantly enhance their immunomodulatory potency. However, clinical translation faces significant hurdles, including incomplete structure-activity understanding, undefined pharmacokinetics, low oral bioavailability, challenges in quality control, and a paucity of systematic clinical safety and efficacy data. This review provides a comprehensive synthesis to inform future research and support the potential development of CYPs as novel immunomodulatory agents.

## 1 Introduction

The immune system stands as a fundamental pillar of host defense, maintaining homeostasis and providing protection against pathogens and malignant cells (1). However, its dysregulation is a hallmark of numerous diseases. This dysfunction manifests across a spectrum: immunodeficiency states, characterized by a deficient or absent immune response, lead to heightened susceptibility to recurrent and chronic infections; conversely, the breakdown of self-tolerance results in autoimmune disorders, where the immune system erroneously attacks the body’s own tissues. Furthermore, dysregulated, non-resolving inflammation is central to a wide array of inflammatory conditions. In the context of cancer, an inadequate or suppressed anti-tumor immune response often underlies the failure of immunotherapy, such as checkpoint blockade (2, 3). Consequently, the pursuit of safe and effective agents capable of fine-tuning immune responses immunomodulators remains a paramount objective in biomedical science. In this review, “immunomodulatory effect” refers to the bidirectional or homeostatic regulation of the immune system. This encompasses the potential to enhance immune responses, such as stimulating macrophage phagocytosis, lymphocyte proliferation, and antibody production under conditions of immunosuppression or immunodeficiency, as well as the capacity to temper excessive or aberrant inflammation, thereby contributing to the restoration of immune equilibrium. It is distinct from a mere potentiation of the inflammatory response. In this quest, natural products, particularly those with a history of medicinal and dietary use, have attracted sustained interest due to their perceived safety, structural diversity, and multi-target potential (4). Among these, polysaccharides derived from plants, fungi, and other natural sources have emerged as a promising class of bioactive compounds, demonstrating a remarkable ability to influence both innate and adaptive immunity through interactions with various immune cells and pathways (5–7). Despite this promise, the translation of natural polysaccharides from promising laboratory findings to clinically validated therapeutics faces significant and common hurdles. A critical barrier is the frequent incomplete characterization of their complex, heterogeneous structures (8). Bioactivity is not a simple function of monosaccharide units but arises from a precise hierarchical organization encompassing molecular weight, linkage patterns, branching, and three-dimensional conformation (9). The frequent lack of defined structure-activity relationships (SAR) for many polysaccharides obscures which structural features drive specific immune effects. Furthermore, while immunomodulatory effects are often reported, the precise molecular mechanisms, including the initial receptor interactions on target cells and the ensuing intracellular signaling cascades are frequently inadequately elucidated (10, 11). This mechanistic ambiguity, coupled with challenges in standardizing batches, undefined pharmacokinetics, and a paucity of systematic clinical trial data, creates a formidable “translational valley of death” for many bioactive polysaccharides.

Among the diverse array of bioactive plant polysaccharides, those extracted from Chinese yam (*Dioscorea opposita* Thunb), a traditional medicinal and edible plant, have attracted significant research interest (12, 13). Chinese yam polysaccharides (CYPs) are regarded as a principal bioactive fraction responsible for the plant’s multifaceted health benefits, with immunomodulation being a prominent and well-documented effect (14). Extensive preclinical evidence demonstrates that CYPs can activate innate immune sentinels such as macrophages and dendritic cells (DCs), modulate the proliferation and functional differentiation of T and B lymphocytes, and engage in cross-talk with the gut microbiota to exert systemic immunoregulation (15–17). These properties position CYPs as compelling candidates for development as immune-supportive nutraceuticals or novel therapeutic agents. Nevertheless, the research landscape for CYPs reflects the broader field’s challenges. Reported immunomodulatory activities, while robust, are underpinned by a fragmented understanding. The structural code governing CYP bioactivity is not fully deciphered; preparations are typically heterogeneous, exhibiting wide molecular weight distributions and variable monosaccharide compositions influenced by botanical source and extraction methods (18, 19). Although Toll-like receptor 4 (TLR4) has been implicated as a key pattern recognition receptor (20–22), a comprehensive SAR framework linking specific structural features to defined immune outcomes is still evolving. Most critically, the transition from convincing laboratory and animal model data to human application remains virtually unexplored, with no registered clinical trials specifically evaluating CYP’s immunomodulatory efficacy. Consequently, a systematic and critical synthesis that examines the chemical foundations of CYP activity, consolidates mechanistic insights, and rationally analyzes translational barriers is essential to guide future research and potential development.

This review directly addresses these identified scientific and translational gaps. We present a comprehensive analysis that traverses the journey from the structural underpinnings of CYPs to their immunomodulatory mechanisms and the hurdles of clinical application. Specifically, we systematically consolidate current knowledge on the key chemical-structural features of CYPs, including molecular weight, monosaccharide profile, glycosidic linkages, and spatial conformation and critically evaluate their established correlations with immunologic activity. We elucidate the molecular mechanisms of action, with a focus on TLR4-dominated signaling and the consequent activation of macrophages, DCs, and lymphocytes, alongside the immunologically significant interplay with the gut microbiota. Furthermore, we evaluate bio-enhancement strategies such as chemical modification and nano-formulation. Finally, we provide a candid appraisal of the major barriers to clinical translation encompassing structural heterogeneity, undefined pharmacokinetics, and a lack of clinical evidence and propose a strategic roadmap for future research aimed at bridging the divide between the preclinical promise of CYPs and their potential realization as novel immunomodulatory agents.

## 2 The key chemical structural features of Chinese yam polysaccharides

**Figure 1:**
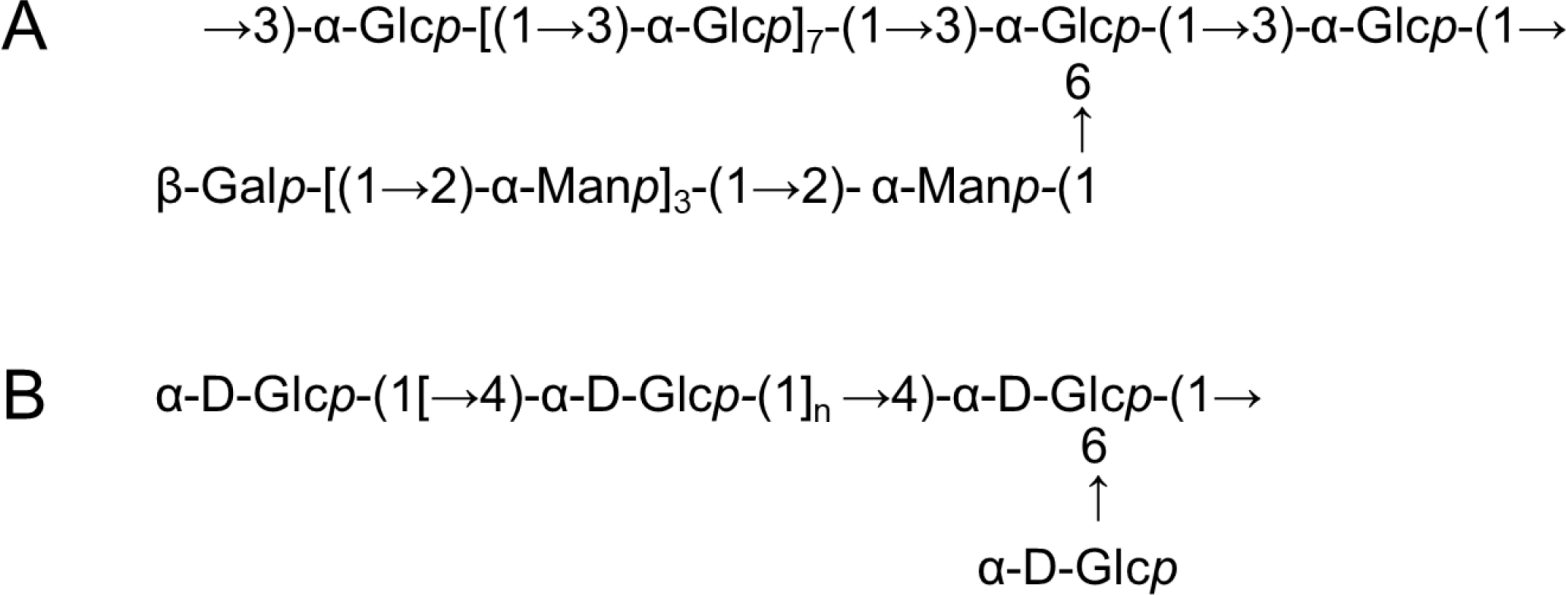
Known structures of yam polysaccharides with immunomodulatory activity. **(A)** Possible structure of YP-1, **(B)** Possible structure of CYP.

The bioactivity of polysaccharides is not simply determined by their monomers but is collectively governed by a series of complex structural factors, including degree of polymerization, monosaccharide composition and ratios, glycosidic linkage patterns, branching degree, spatial conformation, and functional group modifications. For Chinese yam polysaccharides, the “code” for their immunomodulatory activity is embedded within these precise structural features. As shown in **Table 1**, the main research findings on the structural characteristics of yam polysaccharides with immunomodulatory activity are summarized to provide a comprehensive overview. The chemical structures of polysaccharides with reported immunomodulatory activity are presented in **Figure 1**.

**Table 1:** Molecular weight, monosaccharide composition and structural characteristics of yam polysaccharides with immunomodulatory activity.

Name	Molecular weight(kDa)	Monosaccharide composition	Structural characteristics	Analytical techniques	Ref
CYP-LS	34.111	Rha: Ara: Gal: Glu: Man= 0.2849:0.182:0.5684:1.4069:3.7227	–	HPAEC, HPSEC, FT-IR	(23)
CYP-NF	124.774	Rha: Ara: Gal: Glu: Man= 0.493:0.6695:0.9738:0.7655:12.4365	–	HPAEC, HPSEC, FT-IR	(23)
YP-1	42	Glu: Man: Gal= 1:0.37:0.11	(1/3)-linked a-D-glucopyranosyl residues	HPSEC, GC–MS, FT-IR, NMR	(17)
CYP	19.5	Gal: Glu: Xyl: Ara: Rha: GalA= 1.41:0.98:0.91:0.27:0.18:2.77	–	HPAEC, HPLC	(24)
S-CYP	29.6	Gal: Glu: Xyl: Ara: Rha: GalA= 2.82:0.65:1.97:0.76:0.12:1.99	–	HPAEC, HPLC	(24)
YPS	–	Fru: Xyl: Gal: Man: Glu: GalA= 0.59:1.33:8.06:9.00:34.94:35.82	–	GC–MS	(25)
SeYPS-1	–	Fru: Xyl: Gal: Man: Glu: GalA= 0.58:1.58:3.57:7.78:8.92:35.38:35.66	–	GC–MS	(25)
SeYPS-2	–	Fru: Xyl: Gal: Man: Glu: GalA= 0.55:1.39: 8.15:10.51:33.96:36.23	–	GC–MS	(25)
CYP	33.33	Rha: Gal: Glu: Xyl: GalA: GluA= 1.77:11.36:13.44:1.53:15.47:1.67	–	HPAEC, HPLC, FT-IR, SEM	(26)
S-CYP	37.04	Rha: Gal: Glu: Xyl: GalA: GluA= 0.13:13.51:12.98:1.25:16.22:0.91	–	HPAEC, HPLC, FT-IR, SEM	(26)
YPs	2.06-2.28	Man: GalA: Glu: Gal: Ara=0.47: 0.86: 1.00: 1.13: 1.24	–	HPGPC, FT-IR	(27)
DOP-2	1.52×10^3^	Glu: Gal: Ara= 26.12: 53.45: 20.43	→3,6)-βD-Galp (1→	HPGPC, HPLC, SEM, GC -MS	(28)
CYP	9.931	Glu: Gal= 98.87: 1.13	→4)-α-D-Glcp-(1→	GPC, HPAEC, FT-IR, XRD, NMR	(29)
CYPP-1	50.5	Glu: Gal: GluA: Man: Ara= 59.4:1.0:0.18:0.13:0.13	–	HPGPC, HPLC, FT-IR	(30)
CYPP-1	4.4	Glu: Gal: GluA: Man: Ara= 59.4:1.0:0.18:0.13:0.13	–	HPGPC, HPLC, FT-IR	(30)
CYPP-HWP	168.441	Ara: Gal: Glu: Man= 2.61: 6.41:531.51:13.94	–	HPAEC, SEC, SEM	(31)
CYPP-USP	344.777	Ara: Gal: Glu: Man=2.41: 9.42:492.39:22.50	–	HPAEC, SEC, SEM	(31)
CYPP-HHPP	237.056	Ara: Gal: Glu: Man=2.17:6.29:522.78:16.47	–	HPAEC, SEC, SEM	(31)
CYPP-PEFP	183.117	Ara: Gal: Glu: Man=2.96:8.11:482.42:20.15	–	HPAEC, SEC, SEM	(31)
CYPP-CPP	207.039	Ara: Gal: Glu: Man=2.57:4.79:512.64:15.78	–	HPAEC, SEC, SEM	(31)
CYPP-MWP	353.350	Ara: Gal: Glu: Man=2.98:6.93:509.19:16.22	–	HPAEC, SEC, SEM	(31)
CYPP-RFP	305.750	Ara: Gal: Glu: Man=2.96:5.51:503.86:17.81	–	HPAEC, SEC, SEM	(32)
NSCYP	71.78	Glu: Gal: Man: Ara: GalA= 1.0:3.03:1.09:1.77:0.51	1,4-Galp, 1,5-Araf, 1,4-Glcp, 1,4- GalAp, 1,4- GalMEp, 1,6-Galp, 4,6-Galp, 4,6- Manp and t-Araf, tGalp, t-Glcp, tGalMEp, t-GalAp	HPSEC–MALLS–RID, HPLC, GC–MS	(33)

Mannose (Man), Rhamnose (Rha); Galactose (Gal); Glucose (Glu); Xylose (Xyl); Galacturonic acid (GalA); glucuronic acid (GluA); Fructan (Fru).

### 2.1 Molecular weight

The molecular weight (MW) of Chinese yam polysaccharides represents a critical physicochemical parameter, as it directly governs their solubility, rheological properties, and consequent bioactivity. Research consistently demonstrates substantial molecular weight heterogeneity and pronounced polydispersity in yam polysaccharides, attributable to variations in plant species, geographic source, and extraction/purification protocols. The standard isolation process typically involves hot-water extraction, with subsequent ethanol precipitation, deproteinization, and decolorization to yield a crude polysaccharide preparation. Given the inherent heterogeneity of the crude extract, further purification techniques are routinely applied to isolate polysaccharide fractions with specific molecular weight ranges. Commonly used purification methods include ion-exchange chromatography and size-exclusion chromatography (34, 35). By leveraging differential molecular size and charge, these chromatographic methods enable the separation of polysaccharide mixtures into multiple, more homogeneous components. For instance, Li and his research team (36) successfully separated yam polysaccharides into fractions with different molecular weights, such as >50 kDa, 10-50 kDa, and <10 kDa components, for subsequent comparative activity studies. Accurately determining the molecular weight of these fractions is a fundamental step in characterization. High-Performance Gel Permeation Chromatography (HPGPC) is now the most prevalent and robust methodology for this purpose. HPGPC determines the Mw and number-average (Mn) molecular weights, along with the polydispersity index (PDI = Mw/Mn), by calibrating the column with standards of known molecular weight and comparing sample retention times. A PDI near unity reflects a narrow, homogeneous molecular weight distribution, whereas a PDI >1 indicates a heterogeneous mixture of chains with different lengths (37–40). Applying these techniques has revealed that yam polysaccharides exhibit a broad molecular weight range, from several thousand to several million Daltons, with reports extending to tens of millions (33). For example, fractions with low molecular weights, such as 2.86 kDa (32), 4.2 kDa, and 4.4 kDa (41), have been isolated. However, the majority of research has concentrated on polysaccharides in the tens to hundreds of kilodalton range, such as a fraction of 20.89 kDa (42) and a component named DOTP-80 with a molecular weight of 123 kDa (43). A significant analytical consideration is that yams contain abundant starch, which is characterized by a very high molecular weight (4.416 × 10^7^ to 8.353 × 10^7^ g/mol) (44). Therefore, effective separation and purification to remove contaminating starch is imperative in yam polysaccharide research to guarantee the study targets are the immunomodulatory non-starch polysaccharides.

In summary, a broad molecular weight distribution and inherent polydispersity are hallmark features of yam polysaccharides (45). This heterogeneity presents a major challenge for establishing clear structure-activity relationships, as bioactivity data from such mixtures cannot be attributed to a specific MW. Consequently, future SAR studies must prioritize the isolation and exclusive use of fractions with narrow, well-defined MW ranges to accurately delineate the correlation between molecular weight and immunomodulatory potency.

### 2.2 Monosaccharide composition

Monosaccharide composition is the most fundamental structural parameter of a polysaccharide, providing the chemical basis for the structural and functional diversity observed in these biopolymers. While earlier work often characterized Chinese yam polysaccharides as glucan-dominated (46), contemporary research has uncovered significant complexity and heterogeneity in their monosaccharide profiles. Analyses employing techniques like GC-MS and HPLC demonstrate marked differences in the monosaccharide composition of yam polysaccharides derived from different sources or purification fractions. As an illustration, Feng et al. (47) isolated and purified an acidic polysaccharide, designated CYP, from Chinese yam. Compositional analysis indicated that CYP consisted of rhamnose (Rha), arabinose (Ara), galactose (Gal), glucose (Glc), xylose (Xyl), galacturonic acid (GalA), and glucuronic acid (GlcA), with a molar ratio of 3.79:9.17:16.68:18.81:30.24:3.30:1.00, where Xyl was the predominant monosaccharide. In a separate study, Bai et al. (48) isolated polysaccharides from the tuber (CYP) and peel (CYPP) of a specific Chinese yam cultivar using hot water extraction, enzymatic hydrolysis (thermostable α-amylase, papain, amyloglucosidase), and JK008 macroporous resin purification. Their analysis showed both fractions contained Man, Gal, GalA, Ara, Glc, Rha, and GlcA, but with distinct molar percentages of 29.72:21.77:28.45:8.14:6.36:3.64:1.96 for CYP and 57.81:18.80:6.20:7.78:4.60:3.48:1.35 for CYPP. These examples collectively indicate that yam polysaccharides are heteropolymers rather than simple glucans, a complexity that underpins their diverse biological activities. This observed heterogeneity arises from multiple factors. Prior research confirms that yam cultivar, geographic origin, growth period, and the specific extraction and purification protocols employed all significantly impact the resulting polysaccharide’s monosaccharide profile (42). For example, polysaccharides from different Dioscoreaspecies, such as Chinese yam (*Dioscorea opposita*), Japanese yam (*Dioscorea japonica*), and Korean yam (*Dioscorea batatas*), are likely to possess distinct structural features (49–51). A comparative study of polysaccharides from three rhizomatous plants, including the related genus *Polygonatum*, revealed similar monosaccharide types but differing proportional contents (52). Among these factors, the choice of extraction and purification techniques represents the most significant experimental variable. Zhu et al. (53) isolated three polysaccharides (CYZ, CYS-1, CYS-2) from Huaishan yam using hot water extraction, with subsequent purification by DEAE-52 cellulose and Sephadex G-100 chromatography. Monosaccharide analysis indicated CYZ contained Man, Glc, Gal, Xyl, and Ara in a molar ratio of 1:13.057:26.56:6.07:2.22, whereas CYS-1 and CYS-2 both consisted of Man, Rha, GlcA, Glc, Gal, Xyl, and Ara, with molar ratios of 1:0.024:0.05:0.084:2.59:0.13:0.14 and 1:0.82:3.86:2.68:12.88:1.29:0.54, respectively. Similarly, Lu et al. (31) compared polysaccharides extracted from yam peel using hot water (HWP) versus six physical-assisted techniques: ultrasound (USP), high hydrostatic pressure (HHPP), pulsed electric field (PEFP), cold plasma (CPP), microwave (MWP), and radio frequency (RFP). All extracted polysaccharides contained Ara, Gal, Glc, and Man, but the quantitative content of each monosaccharide differed substantially among the extracts. Glucose was the predominant monosaccharide (482.42-531.51 mg/g), whereas arabinose was the minor component (2.17-2.98 mg/g). Compared to conventional hot water extraction, the physical-assisted methods significantly shifted the proportional monosaccharide composition. Beyond extraction, post-isolation processing can also induce structural modifications. Yang et al. (54) demonstrated that fermentation of CYPs using Lactobacillus plantarumM616 significantly modified their chemical properties, including monosaccharide composition. The unfermented (CYP-NF) and fermented (CYP-LP) polysaccharides both contained Rha, Ara, Gal, Glc, and Man, but the molar ratio shifted from 0.493:0.6695:0.9738:0.7655:12.4365 (CYP-NF) to 0.3237:0.3457:0.8278:2.5541:10.4995 (CYP-LP), marked by a pronounced increase in the glucose proportion. This finding offers a novel strategy for the targeted structural and functional modification of yam polysaccharides using biotechnological approaches.

In conclusion, the monosaccharide profile of Chinese yam polysaccharides is characterized by significant complexity and heterogeneity, with no single “standard” ratio. Instead, it is dynamically influenced by biological source and processing methodology. A comprehensive understanding and control of these variables are therefore essential for standardizing production, ensuring quality, and conducting targeted functional research on yam polysaccharides.

### 2.3 Glycoside bond types and main/branch chain structures

Polysaccharides are macromolecular carbohydrates composed of monosaccharide units linked together by glycosidic bonds (39). The diversity in polysaccharide bioactivity fundamentally stems from their structural complexity, which is defined by glycosidic linkage types, monosaccharide composition and sequence, branching architecture, and the resultant three-dimensional conformation (55). Given this complexity, precise structural elucidation is paramount for understanding the bioactivity of yam polysaccharides, with glycosidic linkage analysis representing a crucial aspect of this process.

The anomeric configuration (α or β) of glycosidic linkages is a key determinant of polysaccharide chain conformation and consequent biological function. For instance, β-(1→3)-D-glucans are known for their immunostimulatory properties via specific recognition by the Dectin-1 receptor, while α-(1→4)-D-glucans like starch primarily function as energy reserves (56, 57). Current research on the glycosidic linkage configuration in yam polysaccharides shows some inconsistency, reflecting their structural diversity. Based on FT-IR analysis revealing a characteristic peak at 840-850 cm^-^¹, (43) inferred the presence of α-glycosidic linkages. Supporting this, the presence of α-linkages has been inferred from NMR data in several studies; for example, anomeric proton signals above δ 5.0 ppm in ¹H-NMR or anomeric carbon signals in the δ 90-100 ppm region in ¹³C-NMR are typical indicators of α-configuration (58). Conversely, the presence of β-glycosidic linkages is also reported and considered vital for bioactivity. Ouyang et al. suggested that the prebiotic effect of yam polysaccharides on specific probiotic strains might be linked to β-glycosidic bonds, hypothesizing that the β-configuration could be more favorable for utilization by certain bacteria. It is important to note that while FT-IR and 1D-NMR offer initial insights, the information is macroscopic and represents an average, which is particularly limiting for heterogeneous mixtures. FT-IR peak assignments are empirical, and significant signal overlap in 1D-NMR complicates precise assignment. Therefore, definitive identification of glycosidic linkage configuration and position in a complex polysaccharide requires comprehensive 2D-NMR analysis on a highly purified, homogeneous fraction.

Elucidating the primary structure centers on defining the linear backbone formed by glycosidic linkages between monosaccharide residues and the attachment points of any side chains. This information is fundamental for understanding polysaccharide three-dimensional conformation and molecular recognition patterns. Common backbone motifs in plant polysaccharides include (1→4)-α-D-glucan, (1→3)-β-D-glucan, and (1→4)-β-D-mannan (59, 60). Branch points are commonly at the O-6 position via (1→6) linkages but can also occur at O-2, O-3, or O-4, yielding comb-like or branched architectures (61–63). The definitive methods for determining backbone and branching structures are methylation analysis coupled with GC-MS and comprehensive 2D-NMR spectroscopy. Methylation analysis identifies glycosidic linkage positions and branching points by methylating free hydroxyl groups, whereas hydroxyls involved in linkages remain unmethylated. Complementarily, 2D-NMR, especially HMBC, detects long-range ¹H-¹³C couplings across glycosidic linkages, offering direct evidence for the sequence of monosaccharide residues (64, 65).

Applying these techniques has shown that yam polysaccharide backbones are frequently composed of (1→4)- and/or (1→6)-linked saccharide residues. For instance, Chan et al. used methylation analysis to show a high proportion of 1,4-Glcp residues in a yam polysaccharide fraction, suggesting a backbone potentially resembling either α-(1→4)-glucan or β-(1→4)-glucan (66). Furthermore, yam polysaccharides are widely recognized to exhibit complex branching patterns. Methylation analysis allows for clear identification of branching points; for example, the detection of a (1→3,6)-linked Glcp or Galp residue signifies a branch point where C1 is linked to the preceding residue, and both C3 and C6 are substituted. Methylation analysis also identifies non-reducing terminal residues (67), and the ratio of terminal to branched residues allows estimation of the average chain length and degree of branching—key structural parameters affecting solubility, viscosity, and bioactivity (68, 69). Specific structural models have been proposed. Pan et al. (70) described a yam polysaccharide fraction, DOTP-B, with a proposed backbone of →4)-α-D-Glcp(1→ and →6)-β-D-Galp(1→ (14:1 ratio) and branching at the O-6 of the →4)-α-D-Glcp residues, creating →4,6)-α-D-Glcp branch points. Another study proposed a structure featuring a β-(1,3)/β-(1,6)-galactan backbone with α-(1,4)-Glcp side chains (71).

In summary, while techniques like NMR and methylation analysis are employed, the current structural understanding of CYPs remains fragmented. The proposed models are often tentative due to the analysis of insufficiently purified fractions. A critical limitation is the scarcity of studies on truly homogeneous CYP components, which has precluded the definitive establishment of glycosidic linkage motifs essential for specific immune receptor engagement. Overcoming this requires a paradigm shift towards studying chemically defined, homogeneous polysaccharides obtained through rigorous, multi-step purification.

### 2.4 Advanced structure and spatial conformation

In solution, polysaccharide chains adopt specific three-dimensional conformations, such as random coils, single helices, or triple helices stabilized by secondary interactions including hydrogen bonding, rather than remaining as linear, extended polymers (72, 73). This higher-order structure is recognized as a key determinant of polysaccharide biological function. For example, the renowned triple-helix structure of β-glucans has been confirmed as the structural foundation for their potent immunostimulatory activity (74, 75). Thus, elucidating the advanced structure and spatial conformation of Chinese yam polysaccharides is a prerequisite for understanding their bioactivity and enabling efficient development.

The study of these conformations employs a suite of complementary techniques. Scanning electron microscopy (SEM), while unable to resolve single-molecule conformations in solution, offers valuable insight into the micro-morphology of lyophilized yam polysaccharide powders (76). SEM imaging has revealed distinct morphological features among different fractions, including disordered lamellar aggregates, porous spongy architectures, and complex branched networks (42, 58). Differences in these macro-morphologies indirectly reflect underlying molecular-level variations in composition, linkage conformation, molecular weight, and aggregation propensity.

To probe conformation directly at the molecular level, more advanced techniques are required. Standard methods like FT-IR and UV spectroscopy provide information on functional groups but lack the capacity to directly elucidate three-dimensional conformation (77). In contrast, circular dichroism (CD) spectroscopy is a sensitive tool for probing the existence of ordered secondary structures, such as helices, in polysaccharide chains (78). Atomic force microscopy (AFM) represents a powerful technique for investigating conformation at the nanometer scale. It enables direct imaging of individual polysaccharide chains deposited from dilute solution onto flat substrates like mica, allowing measurement of chain dimensions, branching, and observation of aggregation phenomena (79, 80). Furthermore, its single-molecule force spectroscopy mode allows for the mechanical stretching of individual chains to probe their elasticity and force-driven conformational changes (81, 82). However, direct AFM studies of individual yam polysaccharide chains remain limited, indicating an area for future exploration (83).

For atomic-level structural details, nuclear magnetic resonance (NMR) spectroscopy is paramount. Solution-state NMR (1D/2D) serves as the definitive method for determining primary structure, including sugar ring signal assignment and glycosidic linkage configuration (46, 84). However, signal broadening in solution-state NMR becomes significant for high-molecular-weight or aggregation-prone polysaccharides. This limitation highlights the unique advantages of solid-state NMR (ssNMR), which facilitates atomic-resolution structural analysis of non-crystalline and insoluble solid-state samples (85–87). When coupled with magic-angle spinning (MAS) and multidimensional correlation experiments, ssNMR can define backbone conformation, intermolecular packing, and molecular dynamics (88, 89). Although not yet applied to yam polysaccharides, its success with structural polysaccharides like cellulose and chitin foreshadows considerable potential for this approach.

Cryo-electron microscopy (Cryo-EM) has revolutionized biomacromolecular structure determination, capable of achieving near-atomic resolution three-dimensional reconstructions (90). However, for yam polysaccharides, their inherent flexibility in solution presents a major obstacle for single-particle Cryo-EM analysis. Nonetheless, Cryo-EM would become an indispensable tool if stable, homogeneous polysaccharide assemblies or their complexes with protein receptors can be prepared (91). Given the current experimental challenges associated with some of these techniques, computational approaches such as molecular dynamics simulations and molecular docking have become essential complementary tools for investigating polysaccharide dynamic conformation and interactions (92, 93).

### 2.5 Factors affecting the structure and activity of yam polysaccharides

The biological activity of Chinese yam polysaccharides is intrinsically governed by their chemical structure. Key structural determinants include molecular weight, monosaccharide composition and ratios, glycosidic linkage types and configurations, branching patterns, and three-dimensional conformation. These structural features, in turn, are profoundly influenced by the methods employed for polysaccharide extraction and subsequent processing. (69, 94). The extraction process is the first and most critical step dictating the final structural and functional profile of the obtained polysaccharides. Different extraction principles and conditions differentially affect the efficiency of cell wall disruption, the degree of polysaccharide dissolution, and the potential for degradation or structural modification. Consequently, the choice of extraction method directly impacts not only yield but also the molecular weight distribution, monosaccharide profile, and bioactivity of the final product (95, 96). This is clearly illustrated by comparative studies. For example, polysaccharides extracted from yam peel using hot water (CYPP-1) were primarily composed of glucose and galactose and exhibited antioxidant and immunomodulatory activities (30). In contrast, polysaccharides obtained via deep eutectic solvent extraction contained fractions with distinct glycosidic bond profiles (both α- and β-linkages) and demonstrated pronounced prebiotic effects (58). Beyond extraction, downstream purification techniques are essential for isolating specific structural components associated with targeted bioactivities. The application of techniques like ion-exchange and size-exclusion chromatography enables the separation of heterogeneous crude extracts into more homogeneous fractions. Research utilizing such an approach has successfully linked specific structures to functions. For instance, a purified acidic heteropolysaccharide (CYP, 20.89 kDa) obtained through hot water extraction and chromatographic purification was shown to alleviate oxidative stress in cells via inhibiting the MAPK pathway, an activity linked to its specific monosaccharide composition (predominantly Gal, Glu, and GalA) (42). Similarly, an ultrasonically extracted and purified fraction (CYP-3a) with a unique monosaccharide ratio demonstrated efficacy in alleviating experimental colitis (97).

In summary, the functional output of yam polysaccharides is a consequence of a definable chemical structure, which is meticulously shaped by the entire production pipeline from extraction to purification. Therefore, systematic investigation into the effects of different processing methods on chemical structure is not only crucial for the targeted preparation of polysaccharides with desired functionalities but also serves as the fundamental pathway for elucidating precise structure-activity relationships and promoting their high-value application.

## 3 Molecular mechanisms of regulating innate immunity

**Figure 2:**
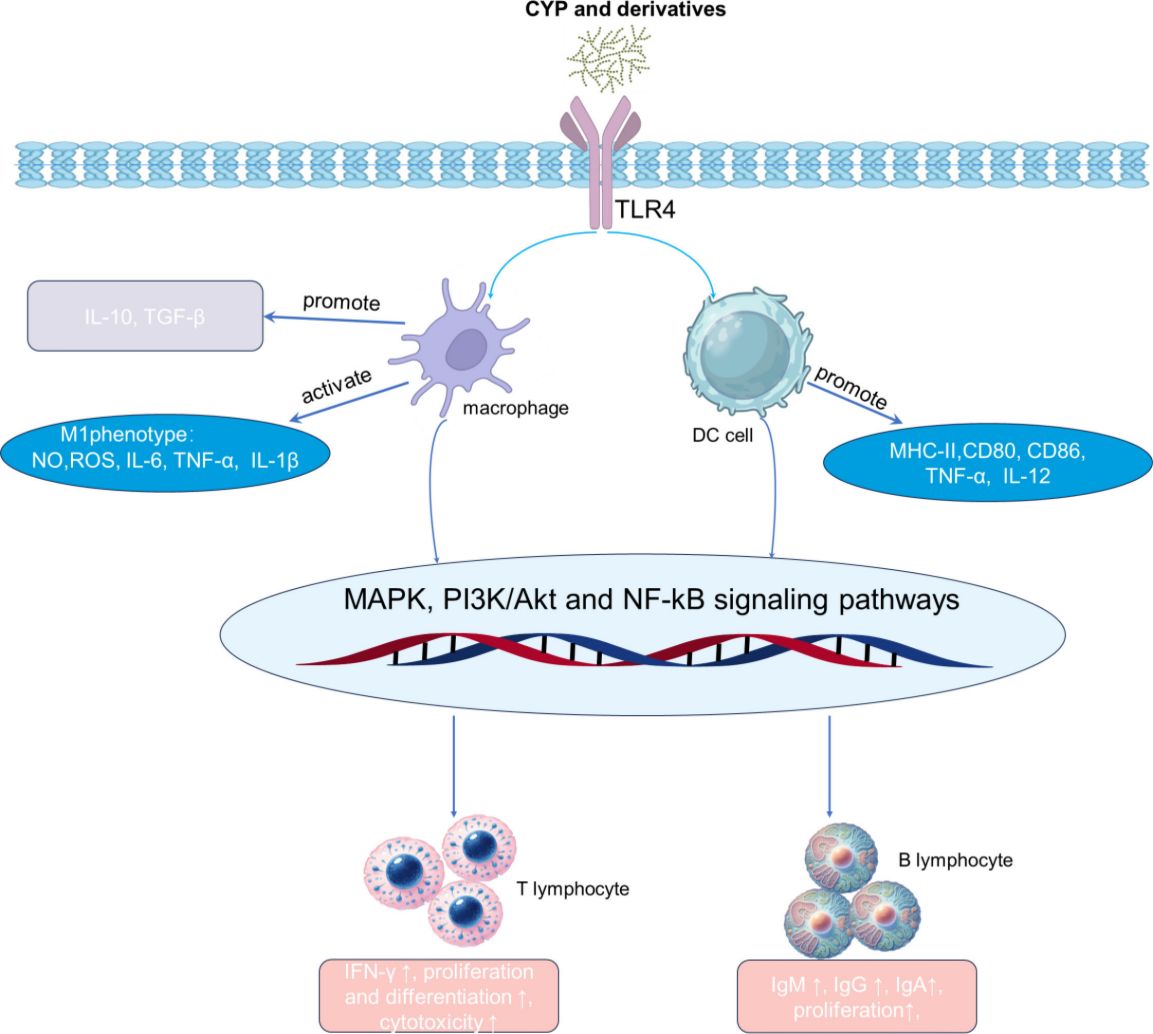
Molecular mechanism of immunomodulation by polysaccharides from yam polysaccharides.

The immune system is a critical defense mechanism that maintains internal homeostasis and protects the host from harmful challenges (98). However, dysregulation of immune function whether manifesting as immunodeficiency or hyperactivation—significantly jeopardizes health by increasing susceptibility to infection or promoting autoimmune and chronic inflammatory disorders. Consequently, identifying safe, effective, and natural immunomodulators is a major focus in pharmacology and functional food science. In this context, plant polysaccharides are recognized as promising candidates due to their broad sources, structural diversity, excellent biocompatibility, and low toxicity (4). Among them, Chinese yam polysaccharides stand out, with their immunomodulatory capacity well-documented in extensive preclinical research (14). Therefore, a central and challenging question, however, is to elucidate the precise molecular mechanisms by which these polysaccharides are recognized by immune cells, the specific signaling pathways they engage, and how they ultimately fine-tune immune responses. A detailed summary of the immunomodulatory activity of yam polysaccharides is presented in **Table 2**, and the proposed molecular mechanisms are illustrated in **Figure 2**.

**Table 2:** Immunoregulation activities of yam polysaccharide.

Polysaccharides	Types	Experimental model	Dosage	Effect	Ref
CYP-LS	*In vitro*	RAW 264.7 cells	5.0, 2.5, 1.25, 0.625, and 0.3125 mg/mL	SOD↑, CAT ↑, GSH-Px↑, TNF-α↑, IL-1β↑, IL-10↑, and TGF-β↑, MDA↓	(23)
CYP-NF	*In vitro*	RAW 264.7 cells	5.0, 2.5, 1.25, 0.625, and 0.3125 mg/mL	SOD↑, CAT ↑, GSH-Px↑, TNF-α↑, IL-1β↑, IL-10↑, and TGF-β↑, MDA↓	(23)
YP-1	*In vivo*	Male Kunming mice	5, 50, 150, and 250 mg/ml	T lymphocytes↑	(17)
SCYP	*In vivo*	Female BALB/c mice	50, 100 and 200 mg/kg	SCFAs↑, trypsin↑, lipase↑, α-amylase↑, *Lactobacillus*↑, *Bacteroidetes*↑, *Akkermansia*↑, *Proteobacteria*↓, *Verrucomicrobia*↓	(99)
S-CYP	*In vitro*	RAW 264.7 cells	25, 50 and 100 μg/mL	ROS↑, NO↑, TNF-α↑, IL-6↑, TLR4-MAPK/NF-κB signaling pathway	(24)
CYPP-PEI	*In vitro* and *In vivo*	Macrophages and ICR mice	*In vitro*:12.5 µg/mL; *In vivo*: 200 µg/mL	CD80↑, CD86↑, TNF-α↑, IFN-γ↑, and IL-12p70↑ of macrophages, MHCI, MHCII↑, and CD80↑ of dendritic cells, IgG↑	(100)
YPS	*In vivo*	Female BALB/c mice	50, 100, 200, and 400 mg/kg	IgG↑, IgA↑, IgM↑, CD4+ splenic lymphocytes↑, spleen and thymus indices↑	(25)
SeYPS-1	*In vivo*	Female BALB/c mice	100, 200, and 400 mg/kg	IgG↑, IgA↑, IgM↑, CD4+ splenic lymphocytes↑, spleen and thymus indices↑	(25)
SeYPS-2	*In vivo*	Female BALB/c mice	50, 100, and 200 mg/kg	IgG↑, IgA↑, IgM↑, CD4+ splenic lymphocytes↑, spleen and thymus indices↑	(25)
CYP	*In vivo*	Female BALB/c mice	25 mg/kg, 50 mg/kg, 100 mg/kg and 200 mg/kg	CD3^+^CD4^+^ and CD3^+^CD8^+^ T lymphocytes↑, CD4^+^/CD8^+^ ratio↑, TNF-α↑, IL-1β↑, IgG↑, IgM↑, spleen and thymus index↑	(26)
S-CYP	*In vivo*	Female BALB/c mice	25 mg/kg, 50 mg/kg, 100 mg/kg and 200 mg/kg	CD3^+^CD4^+^ and CD3^+^CD8^+^ T lymphocytes↑, CD4^+^/CD8^+^ ratio↑, TNF-α↑, IL-1β↑, IgG↑, IgM↑, spleen and thymus index↑	(26)
CYPP	*In vitro*	Splenic lymphocytes	12.5, 25, 50, 100, 200 µg/mL	T and B lymphocytes cells↑, CD4^+^/CD8^+^ ratio↑	(101)
CYP-PPAS	*In vitro* and *In vivo*	BMDCs and Female ICR mice	*In vitro*:15 µg/mL; *In vivo*: 50 µg/mL	DCs↑, CD4 and CD8 T cells↑, CD4^+^/CD8^+^ ratio↑, IgG↑, IL-6↑, TNF-α↑, IFN-γ↑	(102)
YPL	*In vitro*	Mouse immature DC	15.6, 31.2, 62.5, 125 µg/mL	TNF-α↑, IL-1β↑, DCs↑	(103)
YPs	*In vitro*	RAW264.7 cells	200 µg/mL	RAW264.7 cells survival rate↑	(103)
DOP-2	*In vitro* and *In vivo*	RAW264.7 cells and SPF grade male KM mice	*In vitro*: 10, 50, 100, 250, 500 μg/mL; *In vivo*: 200, 400, and 800 mg/kg/	*In vitro*: NO↑, IL-6↑, TNF-α↑; *In vivo*: spleen and thymus indices↑, IL-2↑, IL-6↑, TNF-α↑, *Bacteroidetes*↑, SCFAs ↑, *Firmicutes*↓, *Akkermansiaceae* ↓,	(28)
CYP	*In vitro*	Mouse peritoneal macrophages	250, 500, 1000 μg/mL	CD80↑, CD86↑, TNF-α↑, IL-1β↑, APC↑, IFN-γ↑, IL-6↑	(29)
CYP	*In vivo*	Male C57BL/6 mice and BALB/c mice	100mg/kg	*Clostridia_UCG-014*↑, *Actinobacteria*↑, CD8^+^ T↑, granzyme B↑, 3-indoleacetic acid↑, indole-3-ethanol↑, 5-methoxytryptamine↑, Taurine↑, *Enterorhabdus* ↓, *Desulfovibrionaceae*↓, CD206^+^↓, Deoxyguanosine↓	(104)
CYPP-1	*In vitro*	RAW264.7 cell	10, 40, 80, 120, 160, 200 μg/ml	Phagocytic ability↑, NO↑,	(30)
WYPs	*In vivo*	mice	0, 100 and 500 mg/kg	spleen and thymus indices↑, Phagocytic ability↑, IL-2↑, IFN-γ↑, IL-1β↑, IL-6↑, TNF-α↑, iNOS↑, lysozyme↑, IgM↑, IgA ↑, IgG↑, NK cell↑	16

(Increase, ↑; Decrease, ↓).

### 3.1 The dominant role of toll-like receptor 4

Immune cells sense diverse molecular signals via pattern recognition receptors (PRRs) on their surface. The structural motifs of plant polysaccharides, such as those from Chinese yam, can mimic microbial components and are recognized by PRRs, thereby activating innate immunity. Among these receptors, TLR4 is established as a pivotal and dominant receptor for transducing the immunomodulatory effects of many plant polysaccharides (105, 106). Accordingly, for Chinese yam polysaccharides, a substantial body of evidence indicates their immunomodulatory action is largely mediated through TLR4 activation, which initiates downstream signaling cascades including NF-κB, MAPK, and PI3K/Akt pathways (107).

Direct evidence for TLR4 dependency comes from receptor-blocking studies. Li et al. (33) demonstrated that neutralizing TLR4 significantly abrogated the capacity of a Chinese yam non-starch polysaccharide (NSCYP) to activate macrophages, an effect shown to be mediated via the TLR4/NF-κB signaling pathway. Mechanistically, the binding of yam polysaccharides to macrophage surface TLR4 leads to the recruitment of adaptor proteins, primarily activating the MyD88-dependent pathway (108, 109). This activation in turn triggers a cascade involving TNF receptor-associated factor 6 (TRAF6) and the TGF-β-activated kinase 1 (TAK1) complex. Activated TAK1 then phosphorylates and activates both the MAPK family members (p38, ERK, JNK) and the IKK complex (110, 111).

The activation of these kinases converges on key transcription factors. Specifically, IKK activation leads to the phosphorylation and degradation of the NF-κB inhibitor IκBα, which allows the released NF-κB to translocate to the nucleus. This mechanism is supported by Western blot and immunofluorescence analyses, which confirmed NSCYP-induced IκBα degradation and p65 nuclear translocation in macrophages (33). Concurrently, MAPK pathway activation induces the transcription factor AP-1 (comprising c-Fos and c-Jun). The coordinated action of nuclear NF-κB and AP-1 drives the transcription of immune-related genes, such as pro-inflammatory cytokines (TNF-α, IL-6, IL-1β) and iNOS (112).

The functional significance of the polysaccharide-TLR4 interaction is further underscored by structure-activity relationship studies. Liu et al. (24) demonstrated that sulfation modification markedly potentiated the immunomodulatory activity of Chinese yam polysaccharides. Mechanistic investigation revealed this enhancement correlated with the increased affinity of the sulfated polysaccharide for TLR4, leading to more potent activation of the downstream TLR4-MAPK/NF-κB signaling cascade. These findings collectively imply that chemical modification can optimize polysaccharide-TLR4 interactions. While TLR4 plays a dominant role, interactions with other PRRs remain plausible. For example, Toll-like receptor 2 (TLR2) recognizes bacterial peptidoglycan, and certain polysaccharide structures have been reported as TLR2 ligands (113, 114). Moreover, based on their monosaccharide composition, C-type lectin receptors (CLRs) like Dectin-1 and the mannose receptor represent additional potential targets (115). Therefore, it is likely that different polysaccharide fractions from Chinese yam exhibit varied receptor affinity and selectivity, a prospect that requires further investigation.

### 3.2 Activation of macrophages

As versatile effector cells of the innate immune system, macrophages play a pivotal role in pathogen clearance, antigen presentation, tissue repair, and the regulation of inflammation. Therefore, polysaccharide-mediated macrophage activation is a key immunomodulatory mechanism. Chinese yam polysaccharides can activate resting (M0) macrophages, polarizing them towards a pro-inflammatory M1 phenotype, thereby enhancing their immune surveillance and clearance capabilities (116).

This activation induces a series of characteristic morphological and functional changes. Treatment with Chinese yam polysaccharides leads to classical indicators of an activated state, including cell enlargement, irregular shape, and increased pseudopodia formation. Functionally, this results in markedly enhanced phagocytic capacity, improving the uptake of targets such as neutral red, fluorescent beads, or pathogens (117). Early work by Choi et al. (12) corroborates this, showing that a yam mucopolysaccharide (YMP) could activate mouse peritoneal macrophages by upregulating macrophage survival, enhancing phagocytosis, and promoting lysosomal phosphatase activity. Upon activation, macrophages generate an array of effector molecules. A key antimicrobial mechanism is the production of nitric oxide (NO). Chinese yam polysaccharides potently induce the generation of reactive oxygen species (ROS) and NO in macrophages (118). NO is produced by inducible nitric oxide synthase (iNOS), and these polysaccharides promote its generation by upregulating iNOS expression, constituting a key mechanism for enhancing macrophage cytotoxic capacity. Concurrently, activated macrophages secrete a spectrum of pro-inflammatory cytokines. Chinese yam polysaccharides significantly promote the secretion of cytokines including TNF-α, IL-1β, and IL-6 (119a; 120). These cytokines act in concert to recruit more immune cells to the site of infection, increase vascular permeability, and activate other immune cells, thereby amplifying the local inflammatory response to eliminate pathogens.

In summary, through this multi-faceted activation of macrophages encompassing morphological changes, enhanced phagocytosis, and the production of NO and pro-inflammatory cytokines Chinese yam polysaccharides not only strengthen the first line of innate immune defense but also lay the groundwork for initiating an efficient adaptive immune response, thereby demonstrating their potential as broad-spectrum immune modulators.

It is important to note, however, that the immunomodulatory profile of CYPs extends beyond straightforward pro-inflammatory stimulation. While the aforementioned data robustly support their role in activating M1 macrophages, emerging evidence suggests a more complex, context-dependent regulatory capacity. As summarized in **Table 2**, certain CYP fractions can simultaneously induce both classic pro-inflammatory cytokines and key anti-inflammatory or regulatory cytokines in macrophages. This co-induction hints at a regulatory program that may prime the immune system while concurrently activating mechanisms to prevent uncontrolled inflammation. Furthermore, specific structural variants of CYPs have been reported to suppress pro-inflammatory signaling pathways (121, 122). Therefore, the net immunomodulatory outcome may shift from a predominantly stimulatory phenotype towards a more regulatory or resolving phenotype, depending on the specific CYP structure, dose, and the immunological microenvironment. This capacity for bidirectional regulation underscores their true nature as multifaceted immunomodulators.

A crucial caveat in interpreting this mechanistic data is that most studies have utilized heterogeneous CYP preparations. The observed co-induction of opposing cytokines, for instance, could arise from the combined action of multiple polysaccharide species within a mixture, each with distinct bioactivities, rather than from a single molecule’s pleiotropic signaling. Consequently, future work must prioritize correlating specific structural features from such homogeneous fractions with unambiguous signaling outcomes (e.g., NF-κB versus MAPK pathway bias) in standardized assays to truly decipher the structure-activity relationships governing CYP effects on macrophages.

### 3.3 Activation effect on dendritic cells

Dendritic cells (DCs) are the most potent professional antigen-presenting cells, serving as the critical bridge between innate and adaptive immunity (123). Their primary function is to capture, process, and present antigens to naïve T cells, thereby initiating and shaping specific immune responses (124). Therefore, the modulation of DC function represents a pivotal mechanism by which immunomodulators exert their effects.

Chinese yam polysaccharides are hypothesized to activate DCs, largely based on their structural characteristics and analogy to other plant polysaccharides. A key structural feature is the reported high proportion of mannose, suggesting the presence of mannan or glucomannan structures (125). This is significant because DCs express specialized receptors, such as the mannose receptor (MR) and DC-SIGN, which recognize mannose-rich motifs to facilitate antigen uptake and can initiate intracellular signaling (126). Thus, a compelling hypothesis is that Chinese yam polysaccharides bind to these C-type lectin receptors (CLRs) on DCs, promoting endocytosis and intracellular signaling that leads to maturation. Beyond CLRs, Toll-like receptor (TLR) engagement is another plausible mechanism. Although not classic TLR ligands, many plant polysaccharides have been shown to activate immune cells via TLR4 or TLR2. A study on a Dioscorea batatas polysaccharide demonstrated its ability to induce TNF-α production in macrophages via a TLR4-mediated pathway (107). Given that DCs also highly express TLR4, it is likely that Chinese yam polysaccharides can similarly trigger DC maturation through this pathway.

The most direct evidence for DC activation comes from applied research in vaccine adjuvant development. To overcome limitations of traditional soluble polysaccharides, researchers have engineered Chinese yam polysaccharides into nanoparticle (NP) formulations. For instance, CYP-PLGA nanoparticles were shown to be internalized by DCs more efficiently than soluble antigen, an effect attributed to their size and surface polysaccharide promoting receptor-mediated endocytosis (16). More definitively, a study on CYP-aluminum hydroxide nanoparticles (CYP-AlNPs) demonstrated they significantly upregulated maturation markers (MHC-II, CD80, CD86, CD40) and promoted pro-inflammatory cytokine secretion (IL-1β, IL-6, TNF-α) in bone marrow-derived DCs (BMDCs) (127). Mechanistically, this study established that CYP-AlNPs-induced DC activation depended on both the NLRP3 inflammasome and the TLR4-NF-κB signaling pathway.

Despite these promising insights, fundamental research directly linking purified Chinese yam polysaccharides to DC biology remains scarce. Critical gaps persist. Firstly, no study has systematically evaluated the direct effects of well-characterized, purified polysaccharide fractions on DC maturation, cytokine profiles, or T cell polarizing capacity. Secondly, the precise structure-activity relationships are unknown. Finally, while CLR and TLR engagement is hypothesized, the primary receptors and potential synergistic interactions require validation through receptor-blocking or genetic knockout models. To address these gaps and realize their therapeutic potential, a focused research roadmap is needed: 1) Isolate and structurally characterize homogeneous polysaccharide fractions; 2) Systematically assess their effects on DC function using *in vitro* models; 3) Identify the key pattern recognition receptors involved; 4) Elucidate the downstream signaling networks and transcriptional programs they activate.

### 3.4 Lymphocytes

As central players in adaptive immunity, T lymphocytes are vital for host defense against pathogens and tumor cells. The effects of Chinese yam polysaccharides on T cells are characterized by the promotion of proliferation and the modulation of differentiation balance. Early studies established their lymphocyte-activating potential. For instance, Jiang et al. (128) demonstrated that these polysaccharides significantly enhanced the proliferation of mouse splenic lymphocytes, particularly when co-stimulated with T cell mitogens. Subsequent research revealed that this effect is predominantly indirect. Substantial evidence indicates that Chinese yam polysaccharides first act on antigen-presenting cells (APCs), such as DCs and macrophages. By activating receptors like TLR4 on APCs, they promote APC maturation and cytokine secretion, which in turn effectively activates T lymphocytes (129–131). A key immunomodulatory function is the regulation of T helper (Th) cell balance. Th1 cells (secreting IFN-γ) mediate cellular immunity, while Th2 cells (secreting IL-4, IL-10) are involved in humoral and allergic responses. Research suggests Chinese yam polysaccharides can bias the immune response toward a Th1 phenotype. Hao and Zhao (14)reported that they upregulate IFN-γ production while exerting minimal or suppressive effects on IL-4. This Th1-skewing effect, also supported by other studies (132, 133), is critical for potentiating anti-tumor and anti-viral immunity. Chinese yam polysaccharides also potentiate humoral immunity mediated by B lymphocytes. They stimulate B cell proliferation and, *in vivo*, significantly elevate serum levels of key immunoglobulins (IgM, IgG, IgA), thereby bolstering systemic and mucosal antibody defenses (14). These immunomodulatory properties are particularly evident in models of immunosuppression. In cyclophosphamide (CTX)-induced immunosuppressed animal models, yam polysaccharides demonstrate robust immunorestorative effects. They effectively counteract CTX toxicity, reverse immune organ atrophy, and restore compromised cellular and humoral immunity (14, 99), highlighting their potential as protective agents against chemotherapy-induced side effects.

### 3.5 Interplay among yam polysaccharides, gut microbiota, and immunomodulation

The gut microbiota, a vast symbiotic ecosystem, is now recognized as a central regulator of host immune system development and function (134). Dietary polysaccharides that resist host digestion, such as those from Chinese yam, can be fermented by gut microbes, thereby profoundly influencing gut ecology and systemic immunity. This defines Chinese yam polysaccharides as classic prebiotics, whose immunomodulatory effects are increasingly understood to be mediated through complex interactions with the gut microbiota (135, 136).

The prebiotic activity of yam polysaccharides is characterized by selective modulation of microbial composition. Their complex, indigestible structures reach the colon largely intact, becoming a favored substrate for fermentation. Numerous studies show they selectively promote the growth of beneficial bacteria, including classic probiotics like *Bifidobacteriumand* Lactobacillus, as well as key butyrate-producing taxa such as *Lachnospiraceaeand Ruminococcaceae* (137, 138). Concurrently, yam polysaccharide intervention increases microbial alpha-diversity and modulates community structure (beta-diversity), enhancing the overall stability and resilience of the gut ecosystem (139). A primary consequence of this microbial fermentation is the production of immunologically active metabolites, most notably short-chain fatty acids (SCFAs) like acetate, propionate, and butyrate (140, 141). These SCFAs, along with other modulated host metabolites, are critical mediators that link polysaccharide-induced microbial changes to host immune regulation.

This microbiota-mediated mechanism underpins the therapeutic effects observed in various disease models. For example, in metabolic disorder models, yam polysaccharides reverse high-fat diet-induced dysbiosis, increase SCFA-producing bacteria, and improve metabolic parameters, an effect linked to the suppression of dysbiosis-induced inflammation (142, 143). In inflammatory bowel disease, they alleviate colitis symptoms in conjunction with substantial restoration of gut microbial ecology (144). Perhaps most notably, in a colorectal cancer model, yam polysaccharides remodeled the microbiota and metabolic landscape, thereby potentiating the efficacy of anti-PD-1 immunotherapy, highlighting their potential as microbiome modulators in combinatorial therapy (104).

Collectively, the action of CYPs via the gut microbiota exemplifies their capacity for context-dependent immunomodulation. In scenarios of dysbiosis and hyperinflammation, their primary role is to dampen the dysregulated immune response and restore homeostasis. This is distinct from, but complementary to, their direct immunostimulatory effects on immune cells like macrophages and DCs, which may be more prominent in other contexts.

Despite this progress, critical questions remain. The complete spectrum of microbial degradation pathways and metabolites beyond SCFAs has not been fully elucidated. Furthermore, inter-individual variation in baseline microbiota likely leads to heterogeneous responses to intervention. Therefore, addressing these gaps requires future research employing integrated multi-omics approaches and advanced ex vivo models to dynamically map the “polysaccharide-microbiota-host” interactome and pave the way for personalized applications.

## 4 Structure-function relationships in immune regulation

**Figure 3:**
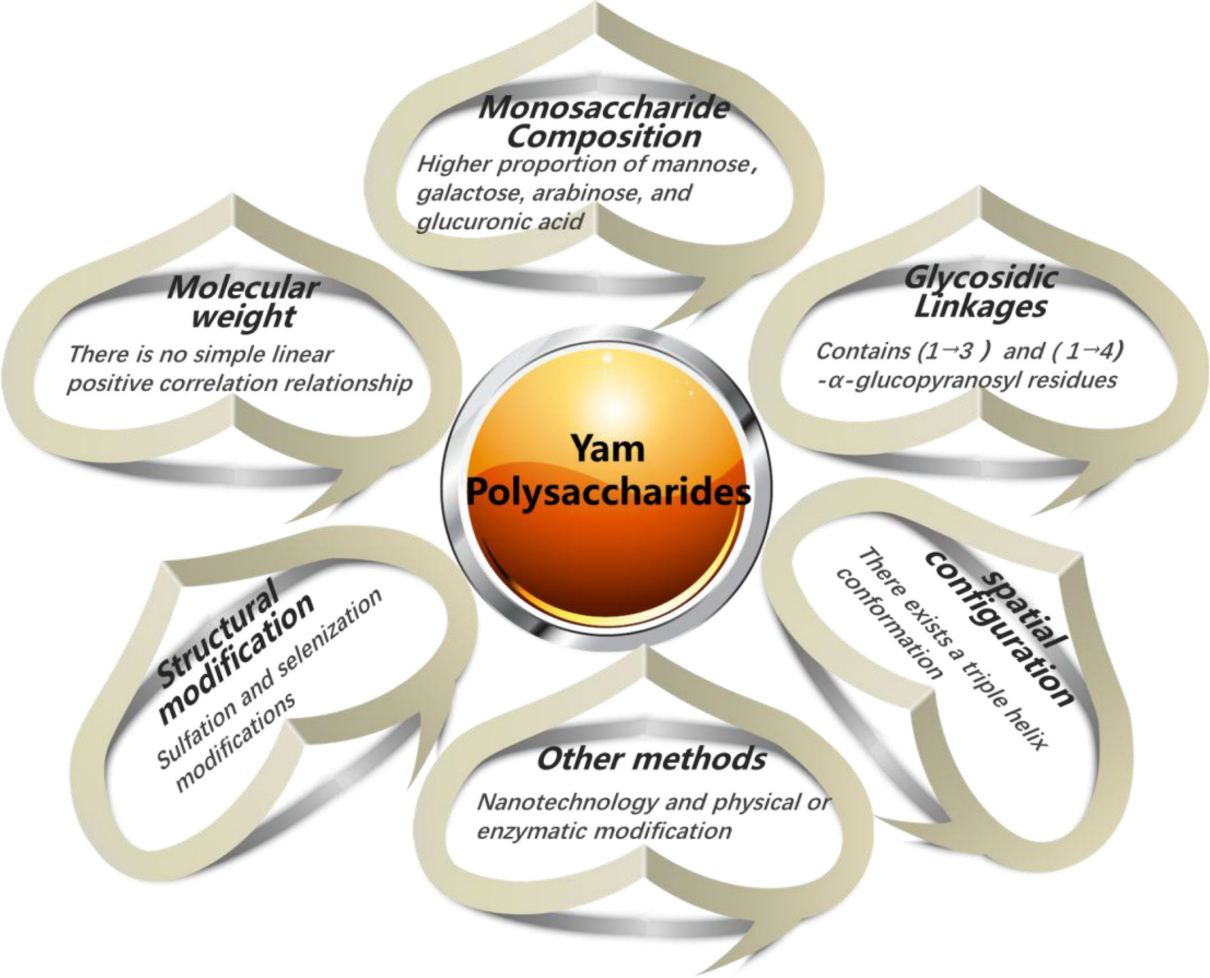
Structure activity relationship of immune regulatory activity of yam polysaccharides.

A fundamental tenet of biology is that the function of macromolecules is dictated by their structure. This principle fully applies to polysaccharides, whose bioactivity arises not from the additive properties of monosaccharide units but from their intricate, hierarchical organization (75, 145, 146). Specifically for Chinese yam polysaccharides, features including molecular weight, monosaccharide composition, glycosidic linkage pattern, branching architecture, and the resultant higher-order spatial conformation all profoundly influence their interactions with biological targets, thereby determining the type and potency of their immunomodulatory activity (58, 147). Therefore, a thorough characterization of these critical structural features forms the essential foundation for mechanistic understanding, structure-activity relationship analysis, and the development of targeted applications. The summary information on the structure-activity relationship of the immunomodulatory activity of yam polysaccharides is shown in **Figure 3**.

### 4.1 Molecular weight

As a fundamental physicochemical parameter, the MW of polysaccharides directly influences their solubility, viscosity, spatial conformation, and ability to interact with cell surface receptors, thereby determining their bioactivity (148). The relationship between MW and activity is complex and non-linear, often operating within an “optimal activity window.” Excessively high MW can impair solubility and hinder cellular interactions, while excessively low MW may fail to elicit a robust immune response.

Generally, high-MW polysaccharides within the 10-1000 kDa molecular weight range are associated with stronger immunomodulatory effects, such as macrophage activation (149). This is attributed to two key factors. First, their longer chains present more repeating motifs, facilitating multivalent binding and cross-linking of receptors like TLR4, which drives receptor clustering and amplifies downstream signaling (150). Second, they can adopt more stable and extended conformations that better present bioactive epitopes to immune cells (151).

Conversely, polysaccharides with MW below 10 kDa often exhibit weaker direct immunostimulation, as they may be too small for effective receptor cross-linking (149). However, lower MW does not inherently equate to lower bioactivity; it may favor different mechanisms. Due to better solubility and permeability, low-MW fractions may be more readily absorbed or exhibit enhanced antioxidant activity, as seen in fermented Chinese yam polysaccharides (152, 153).

Thus, the immunologic activity of Chinese yam polysaccharides is critically dependent on MW, but not in a simple linear fashion. Systematic fractionation of polysaccharides into defined MW ranges, followed by comparative bioactivity screening, is therefore essential to delineate their precise structure-activity relationships.

### 4.2 Monosaccharide composition

The immunomodulatory activity of Chinese yam polysaccharides is critically influenced by their monosaccharide profile. Specifically, polysaccharide fractions with a higher proportion of galactose, arabinose, and glucuronic acid often exhibit stronger immunomodulatory activity (112, 154). Moreover, the presence or relative abundance of trace monosaccharides can be a key determinant of bioactivity (155). A pertinent example is the role of mannose. Immune cells such as macrophages and DCs express surface mannose receptors (MR). Consequently, mannose-rich motifs within yam polysaccharides can engage the MR, triggering downstream signaling that promotes TNF-α, IL-6cytokine release and mediates immune stimulation (117). Therefore, the mannose content and its structural accessibility are considered important factors influencing immunologic activity. This structure-activity principle is exemplified in research on polysaccharides from other medicinal plants, providing a methodological paradigm. For instance, a detailed study on a *Salvia miltiorrhiza* polysaccharide (SMPD-2), which shares a similar monosaccharide profile (arabinose, galactose, glucose), integrated structural analysis with functional assays to demonstrate its potent immunomodulatory and adjuvant efficacy (156). Such an approach, linking precise structural elucidation to immune function evaluation, charts a clear path for future yam polysaccharide research.

While correlations are observed, the evidence remains largely correlative rather than causative. The major confounding factor is that changes in monosaccharide composition (e.g., mannose content) are almost always accompanied by concurrent changes in MW, linkage, and conformation in the compared fractions. Therefore, attributing an immunomodulatory effect solely to one monosaccharide is an oversimplification. Robust SAR claims require studying series of systematically modified, structurally defined analogues where only one structural parameter is varied at a time.

### 4.3 Glycosidic linkages

The immunomodulatory activity of Chinese yam polysaccharides is fundamentally governed by the precise architecture of their glycosidic linkages, including the anomeric configuration (α or β), linkage position (1→3, 1→4, 1→6), and the resulting backbone and branching patterns (157).

Specific linkage types are strongly associated with distinct immunologic effects. Polysaccharides featuring (1→3)-linked backbones, particularly those with α-configuration, are frequently linked to potent immunostimulation. For example, Zhao et al. (17) demonstrated that the immunostimulatory activity of yam polysaccharide YP-1, which potently enhances ConA-induced T lymphocyte proliferation, is intrinsically linked to its specific structure: a backbone of (1→3)-α-D-glucopyranosyl residues branched with (1→2)-α-D-mannopyranosyl residues terminated by β-D-galactopyranosyl units. Beyond (1→3) linkages, other patterns also contribute significantly. For instance, research by Li et al. (117) showed that an α-1,4-D-glucan motif within a yam polysaccharide activates innate immunity through the macrophage mannose receptor, ameliorates DSS-induced colitis in mice, suppresses NF-κB and NLRP3 inflammasome signaling, and restores the expression of intestinal junction proteins. Thus, the immunologic profile of a yam polysaccharide is dictated by a composite of its specific glycosidic linkage features, which collectively determine its interaction with host pattern recognition receptors and downstream signaling pathways.

### 4.4 Triple helix spatial conformation

The higher-order spatial conformation of polysaccharides is a critical determinant of their biological function, often exerting a more profound influence than primary structure alone (158). Among various conformations, the triple-helix a stable, rigid structure formed by three intertwined polysaccharide chains is particularly noteworthy for its strong association with immunostimulatory activity. Chinese yam polysaccharides have been found to adopt this bioactive conformation. For instance, a polysaccharide designated CIYP possesses a triple-helical structure and exhibits both immunomodulatory and antioxidant activities (159). Mechanistically, CIYP, which features a backbone with diverse (1→6) linkages, exerts its immunomodulatory effect by suppressing the MAPK p38 pathway, leading to the downregulation of key signaling proteins (TAK1, MKK3, p-p38) and pro-inflammatory cytokines (IL-1β, TNF-α, IL-6). It is important to note, however, that the presence of a triple-helix conformation, while highly significant, is not an absolute prerequisite for immunologic activity. Some polysaccharides lacking this specific conformation still demonstrate immunomodulatory properties, indicating that bioactivity arises from a composite of structural factors, with conformation being one important contributor among others (160).

### 4.5 Structural modification

#### 4.5.1 Sulfation modifications

Sulfation is a prominent chemical strategy (161) to enhance and optimize the intrinsic immunomodulatory activity of native CYPs. While native CYPs possess well-documented immunomodulatory properties, sulfation aims to augment their potency and address certain physicochemical limitations. This process entails the introduction of negatively charged sulfate groups onto polysaccharide hydroxyls, structurally emulating natural sulfated glycosaminoglycans like heparin. The introduced charges can increase polysaccharide chain extension, improve water solubility, and modulate interactions with immune receptors such as TLR4, thereby amplifying downstream signaling (162, 163). Consequently, sulfation typically enhances, rather than creates *de novo*, immunomodulatory potency. For CYP, the sulfated derivative (S-CYP) activates macrophages more potently than the native CYP, leading to enhanced phagocytosis, NO release, and production of pro-inflammatory cytokines such as TNF-α and IL-6 (24, 119). Mechanistically, this enhanced activity correlates with more robust activation of the MAPK and NF-κB signaling cascades, likely due to a modulated and amplified interaction with receptors like TLR4 (164). These properties position S-CYP as a promising candidate for developing novel, potent, and low-toxicity immunomodulators of natural origin. Beyond direct immune cell activation, sulfated yam polysaccharides also exhibit gut microbiota-modulating and anti-inflammatory properties, suggesting a broader role in regulating mucosal and systemic immune homeostasis (99, 165). These multifaceted effects position sulfated derivatives as promising candidates for the development of novel immunomodulators.

The potent immunomodulatory activity of S-CYP highlights its considerable application potential. However, the translation of this promising discovery from the laboratory to clinical or commercial products faces significant challenges. A primary hurdle is achieving product standardization and quality control. The inherent heterogeneity of native polysaccharides, compounded by the variable degree of substitution introduced during sulfation, necessitates the establishment of rigorous standards for key structural parameters of both starting materials and final products to ensure batch-to-batch consistency (166, 167). Furthermore, scaling up production presents technical and environmental bottlenecks. Common laboratory-scale sulfation methods involve toxic solvents and are unsuitable for industrial scale-up. Developing green, efficient, and controllable large-scale production processes is therefore a critical need (168). The lack of comprehensive pharmacological and safety data also impedes development. Systematic pharmacokinetic studies are required to understand the absorption, distribution, and half-life of S-CYP, which are essential for rational dosing regimen design (169). Moreover, a full preclinical safety profile, including evaluations of acute/chronic toxicity, immunotoxicity, and genotoxicity is imperative to address potential risks such as reagent residue or cytokine storm (170–172).

In conclusion, sulfation represents a promising and powerful strategy to augment the intrinsic activity of CYPs and improve their drug-like properties, such as solubility and receptor engagement. The development of S-CYP derivatives is motivated by the goal of creating more potent and clinically viable candidates from the native CYP scaffold. However, to realize this translational potential, the significant challenges intrinsic to developing any modified biological—namely standardization, scalable green production, definitive pharmacokinetic studies, and comprehensive safety assessment—must be systematically addressed. Success in these areas would position S-CYP as a high-value derivative stemming from the native yam polysaccharide lead.

#### 4.5.2 Selenisation modifications

Similar to sulfation, selenylation is pursued as a strategy to boost the efficacy and add novel functionalities to the existing bioactivity profile of CYPs. This process involves the covalent incorporation of selenium into the polysaccharide backbone. Selenium is an essential trace element and a cofactor for crucial antioxidant enzymes like glutathione peroxidase (173, 174). The rationale behind selenylation is to create a conjugate that synergistically combines the immunomodulatory framework of the native polysaccharide with the antioxidant and potential immunoenhancing properties of organoselenium. Thus, selenylated derivatives often retain and can significantly amplify the bioactivities of their parent polysaccharides, sometimes displaying potencies superior to those of the native polymer or inorganic selenium alone (175). For CYPs, chemical selenylation effectively enhances immunomodulatory activity *in vitro*. In a foundational study, Guan et al. (15) first applied chemical selenylation to a Chinese yam polysaccharide (YPS), systematically assessing its effect on *in vitro* immunomodulation. Their results showed that a lightly selenylated derivative (SYPS) significantly enhanced RAW264.7 macrophage proliferation compared to the native YPS. Furthermore, SYPS also induced NO production in macrophages more potently than YPS. Furthermore, SYPS treatment led to significantly higher secretion of the pro-inflammatory cytokines TNF-α, IL-6, and IL-1β compared to YPS treatment. These cytokines are vital for immune cell activation and pathogen clearance. Although the specific signaling pathways were not explored in that study, the work by Guan et al. provided the first direct experimental evidence for the feasibility and immune-potentiating efficacy of chemically selenylating yam polysaccharides, offering a preliminary foundation for their development as novel immune-enhancers.

Despite this promising *in vitro* evidence, significant knowledge gaps must be addressed to evaluate the translational potential of selenized CYPs. A primary limitation is that current research is confined to cellular models. It remains to be determined whether these potent effects can be replicated *in vivo*, necessitating systematic animal studies to evaluate oral bioavailability, *in vivo* metabolism, and overall immunomodulatory outcomes in healthy or disease models (176). Furthermore, comprehensive safety assessment is a critical and unexplored area. Selenium is a classic “double-edged sword” element, essential at nutritional doses but toxic at higher levels. The toxicological profile of selenized polysaccharides may differ from that of inorganic selenium. Currently, there is a complete lack of essential toxicology data for selenized CYPs, including assessments of acute, subchronic, and genotoxic effects (177). Addressing these pharmacological and safety gaps is imperative for the rational development of selenized yam polysaccharides.

#### 4.5.3 Other methods for enhancing immune activity potential

Beyond chemical derivatization, strategies such as nanotechnology and physical or enzymatic modification offer alternative routes to enhance the bioactivity and applicability of CYPs. Nanotechnology-based delivery systems present a promising approach to overcome key limitations of native polysaccharides, such as low oral bioavailability and rapid systemic clearance. Encapsulating CYPs within nanoparticles can protect them from premature degradation, enhance their uptake by immune cells, and ultimately improve their bioavailability and immunostimulatory efficiency (178, 179). For instance, encapsulating yam polysaccharides in poly(lactic-co-glycolic acid) nanoparticles (CYP-PLGA-NPs) has been shown to significantly promote lymphocyte proliferation and trigger T helper cell differentiation (101). The nanoparticulate size facilitates uptake by DCs (100). Following internalization, the concurrent slow release of CYP and antigen cargo allows the polysaccharide to activate TLR signaling and promote DC maturation, while the antigen is efficiently processed and presented. This synergistic action can induce a more robust and durable specific immune response compared to soluble antigen alone or a simple mixture of antigen and free polysaccharide (16). The potential of CYP-based nano-adjuvants is further exemplified by composite systems combining CYP with traditional adjuvants. For instance, integrating CYP with aluminum hydroxide creates a novel composite nano-adjuvant (CYP-Al). Preclinical studies across different vaccine platforms validate its efficacy. In an H9N2 avian influenza vaccine model, the CYP-Al formulation induced a balanced and potent antibody response (elevated HI titers, total IgG, IgG1, and IgG2a) and increased splenic CD4^+^ and CD8^+^ T cells compared to aluminum adjuvant alone (180). The molecular basis for this enhanced immunogenicity has been elucidated. In a PCV2 subunit vaccine study, the CYP-Al adjuvant promoted superior antibody responses and germinal center formation. Mechanistically, it was shown to activate dendritic cells via both the TLR4-NF-κB and NLRP3 inflammasome signaling pathways, explaining its ability to synergistically boost both humoral and cellular immunity (127).

In contrast to chemical or nano-modifications, physical and enzymatic methods are considered greener and milder techniques for modification. These methods can cleave polysaccharide chains into smaller fragments, potentially improving water solubility and bioactivity. However, precise control of processing conditions is critical, as excessive treatment may disrupt bioactive conformations and lead to activity loss. Furthermore, understanding the stability of CYPs under physiological conditions is vital for oral applications. Studies simulating gastrointestinal digestion have shown that CYPs can retain a degree of immunomodulatory activity after heat or acid treatment, supporting their potential as oral functional food ingredients (14).

Microbial fermentation is an effective and economical method for structural modification. The process can significantly alter CYP structure, for example, reducing molecular weight and changing monosaccharide composition (181–184). For example, compared to unfermented Chinese yam polysaccharide, the fermented polysaccharide (CYP-LS) exhibited a reduction in average molecular weight from 124,774 Da to 34,111 Da. The molar ratio of glucose increased (from 0.7655 to 1.4069) in CYP-LS, whereas that of mannose decreased markedly (from 12.4365 to 3.7227) (23). Moreover, CYP-LS treatment of RAW 264.7 macrophages enhanced the activities of antioxidant enzymes (superoxide dismutase, catalase, glutathione peroxidase), lowered malondialdehyde levels, and potently stimulated the secretion of TNF-α, IL-1β, IL-10, and TGF-β (23). A combination of CYP-LS and specific probiotic strains (*Lactobacillus helveticus* HH-LPH17, *Lactobacillus johnsonii* 456^®^, and *Lactobacillus acidophilus* HH-LA26) demonstrated synergistic enhancement of immunomodulatory activity. Therefore, microbial fermentation represents a promising method for enhancing the immunomodulatory potential of CYPs. Future research should focus on correlating specific fermentation methods with changes in the physicochemical and biological properties of polysaccharides to guide their industrial application.

## 5 Discussion and prospects

Chinese yam polysaccharides (CYPs) have attracted increasing attention as promising natural immunomodulators because they regulate both innate and adaptive immune responses in a context-dependent manner. The available evidence suggests that their bioactivity is determined not by a single structural feature, but by the combined effects of molecular weight, monosaccharide composition, glycosidic linkage patterns, branching architecture, and higher-order conformation. Among these factors, molecular weight and linkage architecture appear especially important in shaping receptor recognition and downstream signaling events. However, most studies to date have examined heterogeneous fractions, making it difficult to establish direct causal relationships between specific structures and immune outcomes.

Mechanistically, TLR4 is currently the most supported pattern recognition receptor involved in CYP-mediated immunoregulation. CYPs can activate macrophages and dendritic cells and subsequently trigger downstream pathways such as NF-κB and MAPK, leading to cytokine secretion, phagocytosis, antigen presentation, and lymphocyte activation. Nevertheless, TLR4 should not be regarded as the only possible receptor. Other receptors, including C-type lectin receptors and possibly TLR2, may also participate depending on the precise polysaccharide structure. Therefore, future studies should employ receptor-blocking, gene silencing, and knockout approaches to validate receptor specificity more rigorously.

A notable feature of CYPs is their bidirectional immunoregulatory capacity. Depending on structure, dose, and biological context, CYPs may enhance immune activation in immunosuppressed settings or suppress excessive inflammation in inflammatory diseases. This context-dependent behavior is likely influenced by the structural heterogeneity of CYP preparations, the host immune status, and the local microenvironment. Such complexity underscores the need to move from crude extracts to structurally defined, homogeneous polysaccharide fractions.

Another important mechanism involves the gut microbiota. As non-digestible polysaccharides, CYPs can reach the colon and be fermented by intestinal microbes, thereby modulating microbial composition and metabolite production, especially short-chain fatty acids. This microbiota-mediated pathway may contribute substantially to the anti-inflammatory and immunoregulatory effects of CYPs. In disease models such as colitis, metabolic disorders, and cancer immunotherapy, CYPs have shown beneficial effects by restoring gut microbial homeostasis and improving host immune responses. These findings suggest that CYPs function not only as direct immune modulators but also as microbiota-targeted immunoregulatory agents.

Despite encouraging preclinical evidence, clinical translation remains limited. Major barriers include structural heterogeneity, incomplete structure-activity understanding, undefined pharmacokinetics, low oral bioavailability, insufficient safety evaluation, lack of standardized quality control, and scarce clinical evidence. In addition, the use of crude extracts or multi-component herbal formulas makes it difficult to attribute observed effects specifically to CYPs. These issues collectively contribute to a substantial translational gap.

To bridge this gap, future research should focus on purification of homogeneous fractions, systematic structural characterization, multi-omics-based mechanistic analysis, and rigorous receptor validation. In parallel, chemical modification, fermentation, and nano-formulation may help improve potency, stability, and delivery. Standardized pharmacokinetic, toxicological, and clinical studies will also be essential for moving CYPs from laboratory candidates to clinically relevant immunomodulatory agents.

## 6 Conclusion

In summary, Chinese yam polysaccharides are structurally diverse natural macromolecules with significant immunomodulatory potential. Their biological activities are closely associated with molecular weight, monosaccharide composition, glycosidic linkage patterns, and spatial conformation. Current evidence indicates that CYPs exert immune-regulating effects mainly through PRR-related signaling pathways, especially TLR4-mediated cascades, and through interactions with the gut microbiota.

Although preclinical studies have provided strong support for their immune-enhancing and anti-inflammatory activities, clinical translation remains at an early stage. The main obstacles are structural heterogeneity, limited mechanistic clarity, insufficient pharmacokinetic and safety data, and the lack of human clinical evidence. Therefore, future work should emphasize structural standardization, mechanistic validation, and translational studies to support their development as novel immunomodulatory agents.
